# Bats, Bacteria, and Bat Smell V.2.0: Repeatable Sex-Specific Differences in Scent Organ Microbiota

**DOI:** 10.1007/s00248-024-02368-1

**Published:** 2024-03-26

**Authors:** Öncü Maraci, Anna Antonatou-Papaioannou, Sebastian Jünemann, Karin Schneeberger, Michael Schulze, Ingo Scheffler, Barbara A. Caspers

**Affiliations:** 1 Department of Behavioural Ecology, Bielefeld University, Konsequenz 45, 33619 Bielefeld, Germany; 2 Joint Institute for Individualisation in a Changing Environment (JICE), University of Münster and Bielefeld University, Münster, Germany; 3 Evolutionary Biology, Bielefeld University, Universitätsstrasse 25, 33615 Bielefeld, Germany; 4 Institute of Biology-Zoology, Freie Universität Berlin, 14195 Köning-Luise-Str. 1-3, Berlin Germany; 5 Faculty of Technology, Bielefeld University, Universitätsstrasse 25, 33615 Bielefeld, Germany; 6 Institute for Bio- and Geosciences, IBG-5, Research Center Jülich, Bielefeld University, Universitätsstrasse 27, 33615 Bielefeld, Germany; 7 Animal Ecology, Institute of Biochemistry and Biology, University of Potsdam, Maulbeerallee 1, 14469 Potsdam, Germany; 8 Evolutionary Adaptive Genomics, Institute for Biochemistry and Biology, University of Potsdam, Karl-Liebknecht-Straße 24-26, 14476 Potsdam, Germany

**Keywords:** Conceptual replication, Greater sac-winged bats, Sex-specific microbiome, Scent gland microbiota, Olfactory communication, Chemical signalling

## Abstract

Reproducibility is a fundamental principle in science, ensuring reliable and valid findings. However, replication studies are scarce, particularly in ecology, due to the emphasis on novelty for publication. We explored the possibility of replicating original findings in the field of microbial and chemical ecology by conducting a conceptual replication of a previous study analysing the sex-specific differences in the microbial communities inhabiting the wing sacs, a scent organ with crucial functions in olfactory communication, of greater sac-winged bat (*Saccopteryx bilineata*). In the original study, the skin swabs from the antebrachial wing sacs of the males and wing sac rudiments of the females were analysed using culture-dependent methods to test sex-specific differences. The authors demonstrated that males have lower microbial richness and different microbial composition than females. We attempted to reproduce these findings using 16S rRNA sequencing, which offers improved accuracy in pinpointing microbial members than culture-dependent methods because of advanced statistical methods. Our study validated the original study’s findings: Males had a lower microbial richness, and the community composition differed between the sexes. Furthermore, in the current study, males had an increased abundance of bacteria that might potentially be involved in odour production and degradation of malodorous substances and antimicrobial production. Our conceptual replication study corroborated that microbes can play a role in shaping their host’s olfactory phenotype and consequently influence sexual selection. Furthermore, the current study emphasises the importance of replication efforts and hopefully encourages a culture that values replication studies in scientific practice.

**Supplementary Information:**

The online version contains supplementary material available at 10.1007/s00248-024-02368-1.

## Introduction

Reproducibility is a fundamental principle of scientific practice, ensuring the reliability, objectivity and validity of the findings. Replication studies are the cornerstones of reproducibility in terms of testing robustness and should be considered as the safeguard against errors, biases, and even scientific misconduct [1]. Conceptual replication, where the researchers repeat the original study by making deliberate modifications in the methodology to reproduce the findings of the original study [2], has a particular epistemological function: they allow the progression of science in increments, using a more advanced method. Nevertheless, the novelty of the findings has become a prerequisite for publication, making such studies extremely rare, also in ecology [3]. In other words, the scientific community trades off reproducibility with novelty, deepening one of the most prominent problems, the replication crisis [3]. Today, in several fields, including ecology, most of the knowledge on several hypotheses comes solely from first-of-its-kind studies.

One hypothesis which can benefit from validation by conceptual replication is the fermentation hypothesis for chemical recognition, an important concept in olfactory social communication. This theory postulates that odorant molecules produced by symbiotic microorganisms residing in mammalian scent organs as metabolic by-products can contribute to individuals’ scent gland secretions and thereby are consequently involved in chemical signalling [4–6]. Accordingly, mammalian scent organs, being moist, warm, and nutrient-rich, offer a matchless environment for microbial growth [4, 7]. As the composition and structure of these microbial communities are, at least to some extent, determined by host factors such as taxonomy, life-history traits, genetics, and social interactions, microbially produced odours can broadcast complementary information on these underlying host factors [5, 7–9]. Empirical studies have demonstrated that microbially produced odours might encode cues on taxonomic identity [10], sex [11, 12], age [12, 13], group membership [10–12], reproductive cycle [14], and social status [11] of different mammalian hosts.

Scent gland microbiota was also proposed to play a role in the mate choice decisions of a Neotropical bat species, the greater sac-winged bat (*Saccopteryx bilineata*) [15]. *S. bilineata* is an insect-feeding bat species with a harem polygynous mating system [16–18], where a single male defends its harem consisting of up to eight females the whole year [16]. Colonies comprise several harem groups and peripheral males that roost close to the harem territories [16]. The mating season is restricted to a few weeks per year, and females give birth to a single offspring [19]. Although harem males sire more offspring than peripheral males, they do not have exclusive access to the females in their harem and only sire approximately 30% of the young within their territory [20]. The high frequency of extra-harem paternity can be explained by the larger size of the females, which gives them an advantage during agonistic encounters. Consequently, female choice is an essential component of the reproductive ecology of this species, and male fitness depends on advertising their quality [17, 21].

**Fig. 1:**
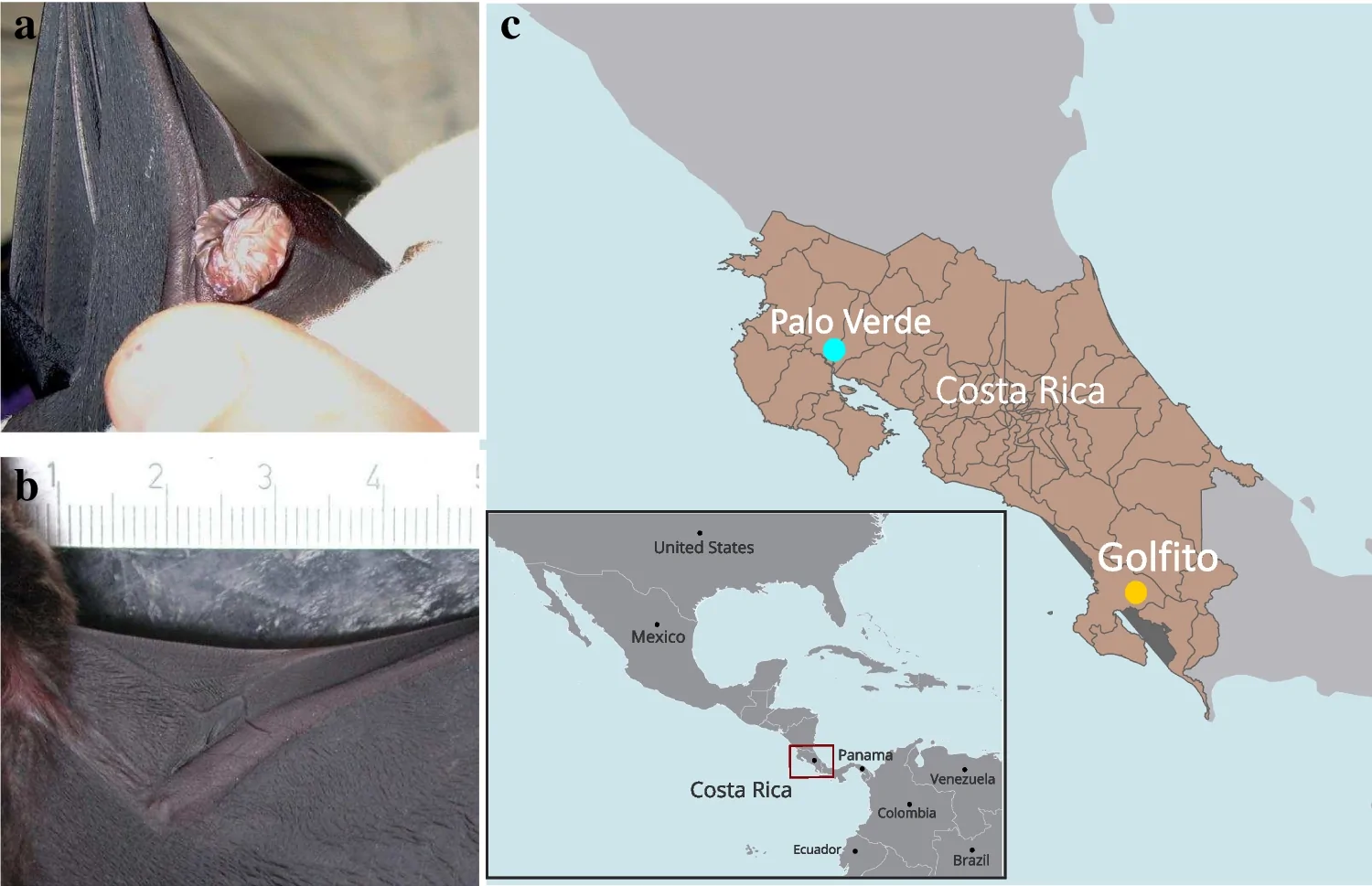
Dorsal view of the antebrachial wing: **a** wing sacs in males, **b** wing sac rudiment of a female, **c** sampling localities

Chemical cues play an important role in the mate choice decisions of this species [18, 22, 23]. Males have pouch-like scent organs in the antebrachial wing (Fig. 1a), which are used to store odoriferous secretions, while females only have the rudiments of these sacs (Fig. 1b) [16]. During courtship, males exhibit hovering flights and fan the odiferous substances from their wing sacs towards females [18, 22]. Wing sac odours carry information on species [24] and individual identity [22], sexual maturity [25], and the geographic distance between colonies [23, 26]. The wing sacs lack glandular tissue and consequently do not produce any secretions [27]. Males clean up and refill these organs every day via a two-step ritual [20, 24, 28, 29]. In the first step, they take up some urine into their mouths and then lick their wing sacs [24, 28–30]. In the second step, they fill the wing sacs with liquids from the genital and gular regions [24, 28–30]. This stereotypic perfume blending behaviour can take up to an hour. It was proposed that males perform this energetically costly and time-consuming behaviour to control microbial growth in the wing sac to minimise microbial fermentation and to generate individual-specific olfactory profiles [15]. Indeed, Voigt and colleagues [15] found that samples originating from the wing sacs of males had lower microbial richness than the samples collected from the wing sac rudiments of females. Undoubtedly providing pioneering insides into the sex-specific alterations in the microbiota of scent organs of *S. bilineata*, the study was conducted using culture-dependent methods (*i.e.* by growing the bacteria in culture media). Today, we know that only a small proportion of the symbiotic microbiota can be cultured, and consequently, relatively recent molecular techniques can provide better resolution in identifying microbial members [9].

Here, we performed a conceptual replication study to characterise wing-sac microbiota collected from two Costa Rican populations of the *S. bilineata*, using a culture-independent molecular method, 16 s ribosomal RNA sequencing, and novel statistical techniques. We evaluated the feasibility of reproducing the findings by Voigt and colleagues [15]. We also tested whether broader identification coverage provided through 16S RNA sequencing can provide a deeper understanding of the sex-specific regulation of the wing sac microbiota.

## Methods

### Sample Collection

Samples used in this study were derived from 56 individuals from two populations (Table 1), Palo Verde National Park (10.378884°N; 85.285158°E) and Golfito (9.588213°N; 83.916920°E) in Costa Rica separated by a distance of approximately 300 km (Fig. 1c), during November and December 2018. The capturing protocol was described by Schneeberger and colleagues [23]. Microbial samples were collected from the area around the wing sacs of males and wing sac rudiments of females using a sterile nylon flocked swab (ESwab, Copan Italia, Italy). Swabs were immediately transferred to liquid Amies medium and stored at − 20 °C. The samples were shipped to Germany on dry ice.

**Table 1:** Samples used in the study

Sex	Population		
	Palo Verde	Golfito	Grand total
Female	4	11	15
Male	22	19	41
Grand total	26	30	56

### DNA Extraction and Library Preparation

Microbial DNA was extracted using BiOstic Bacteremia DNA Isolation Kit (MOBIO Laboratories, Carlsbad, CA, USA) following the manufacturer’s protocol. Subsequently, we amplified the hypervariable V3–V4 region of the 16S ribosomal RNA (rRNA) gene, following the Illumina 16S Metagenomic Library Preparation Guide. The details of the library preparation steps were described by Maraci and colleagues [31]. The two samples were excluded from the analyses due to the unsuccessful amplification after three attempts. The final amplicon pool contained the libraries of 54 biological samples and three blank controls for DNA extraction and amplification, and was sequenced on the Illumina MiSeq system (Illumina, Inc., San Diego, CA, USA).

### Data Analyses

The details of the bioinformatic processing were described in detail by Engel et al. [32]. The processing of raw MiSeq forward and reverse paired-end (PE) reads followed the methodology outlined by Engel et al. [32], with slight modifications and updated versions of utilized tools and databases. Mainly, the paired-end assembly of Miseq PE reads was performed using a custom approach whereby the read pairs were assembled using Flash v1.2.11 [33] in an iterative manner to achieve overall higher assembly rates. Hereby, reads that fail the initial paired-end assembly underwent a 3′ clipping to a q20 average quality threshold using sickle v1.33 [34] before being re-submitted to Flash. This iterative process was continued, incrementally increasing the quality clipping threshold by three, until either all reads were successfully assembled or the maximum quality clipping threshold of q35 was reached. The remainder of the processing steps were conducted as described by Engel et al. [32] with the exception of omitting the length trimming step after primer clipping. In brief, the complete pipeline involved the following steps: paired-end assembly as described above, adapter clipping with cutadapt v1.18 [35], de-replication, alignment to the SILVA seed database v138, filtering off-target aligned reads, and de-noising using mothur v1.41.3 [36], chimera checking, and operational taxonomic unit (OTU) clustering with USEARCH v8.0.1477 [37], and taxonomic classification based on the full SILVA database v138 [38].

All statistical analyses were performed in R version 4.0.0 [39]. We discarded all OTUs classified as archaea, mitochondria, or chloroplasts as an initial quality filtering step. For the alpha diversity analyses, we estimated alpha diversity based on the observed number of OTUs, Chao [32] 1 as the measure of the microbial richness [40], and Shannon’s diversity index, which accounts for both the abundance and evenness of the taxa present [41]. We tested whether the alpha diversity metrics for normality and applied necessary transformation in case of non-normal distribution. Subsequently, we tested whether these metrics differ between sexes and areas using the linear model, using the lm function of R package stats. The residuals of the models were inspected visually.

The compositional differences between sexes were visualised based on the microbial family level taxonomy by stacked bar plots produced by ggplot2 version 3.3.2 [42]. Before beta diversity analyses, we implemented (log10(x + 1)) transformation to deal with unequal sequence coverage. Then, we generated the dissimilarity matrices based on Jaccard, Bray–Curtis, and unweighted UniFrac and weighted UniFrac resemblances. The beta group dissimilarities between sexes were visualised using Principal Coordinate Analysis (PCoA) Plot, implemented using the Vegan package version 2.5–6 [43]. We also statistically tested the differences between samples collected from males and females and different areas by performing a single permutational multivariate analyses of variance (PERMANOVA) [44] model with 9999 permutations. The homogeneity of group dispersions was also tested using PERMDISP, as implemented by the betadisper function in the Vegan package [43]. We also investigated the spatial structuring of the microbial communities by testing the correlations between the distance matrix of the geographical coordinates of the sampling based on the Haversine distances and microbial resemblance matrices using a Mantel test.

The differentially abundant OTUs between sexes were identified using the Corncob package [45], which estimates taxa-specific differential abundances by building beta-binomial regression models, controlling for differential variability across the covariate of interest. We set the significance threshold for *p* values to 0.05 after Benjamini and Hochberg FDR correction [46].

## Results

**Fig. 2:**
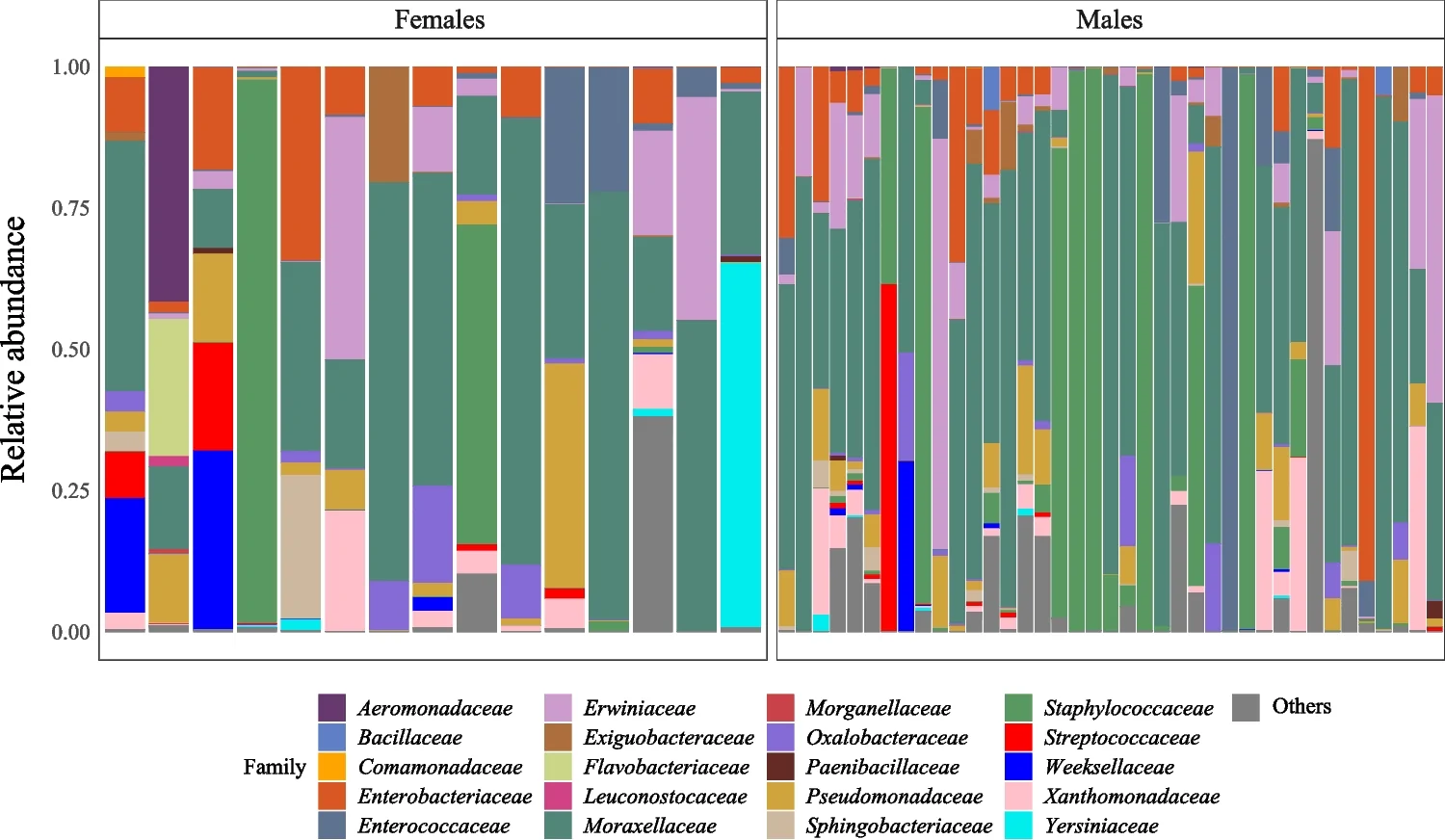
Relative abundance of the 20 most abundant microbial families in wing sac samples of *S. bilineata.* Each stacked bar corresponds to either one female **(a)** or male **(b)** sample. The remaining microbial families are pooled as “Others”

We sequenced the hypervariable V3–V4 region of the 16S rRNA gene from wing sac swabs originating from 56 bats (41 males, 15 females) from two different Costa Rican populations (Table 1). After OTU filtering and excluding two samples due to unsuccessful amplification, our dataset consisted of 54 samples (39 males, 15 females) and 277 different operational taxonomic units (OTU), with a total read count of 4,089,801. We identified seven microbial phyla, with the domination of Proteobacteria (78.23%), Firmicutes (15.63%), and Bacteroidota (6.11%). At a finer taxonomic scale, identified taxa corresponded to 74 microbial families (Fig. 2).

## Sex-Specific Differences in Microbial Richness, Diversity, and Composition

The numbers of females and males, retained in our final dataset, were 15 and 39, respectively. Samples collected from females contained, on average, 43.2 individual OTUs (minimum 19; maximum 71; SD 14.21) with an average read count of 107,896.2 (SD 103,380.93). Samples collected from males contained, on average, 33.5 individual OTUs (minimum 14; maximum 75; SD 11.8) with an average read count of 63,368.15 (SD 93,468.92).

**Fig. 3:**
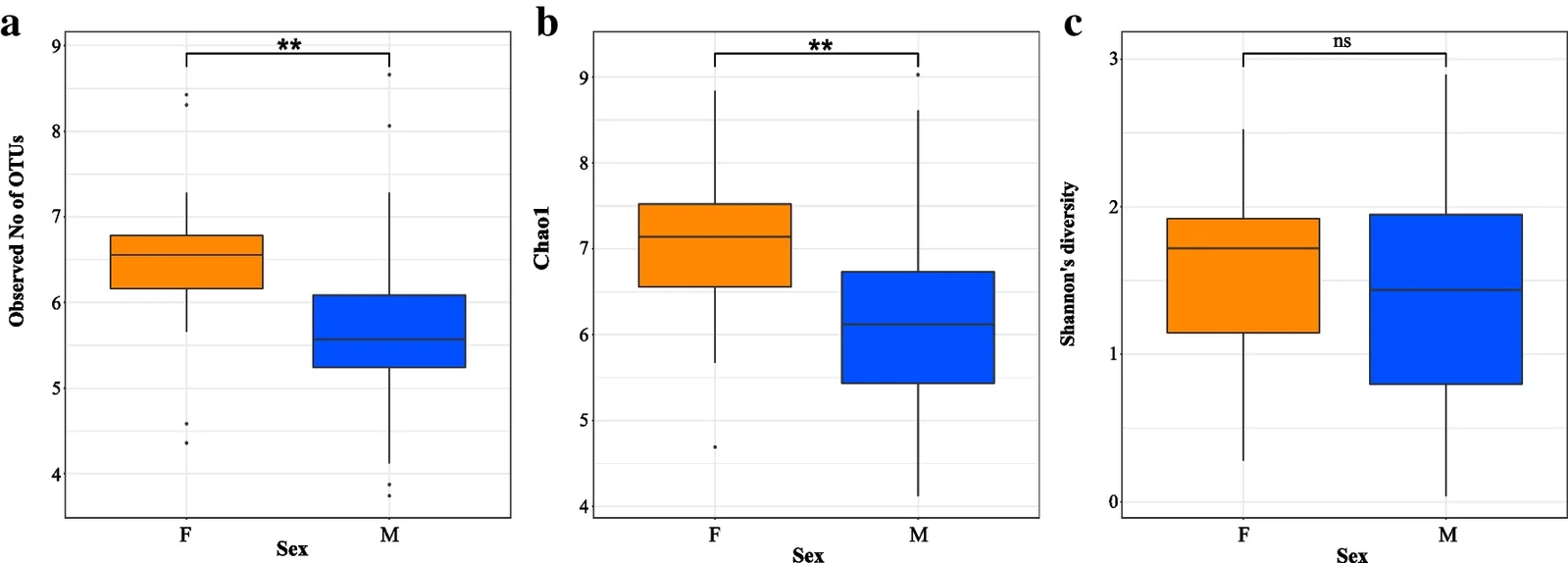
Sex-specific differences in alpha diversity metrics. Sex differences in **a** the observed number of operational taxonomic units, **b** Chao 1, and **c** Shannon’s diversity index. The significant differences were determined based on the linear mixed model at *p* values ≤ 0.05 (*), *p* ≤ 0.01 (**), and *p* ≤ 0.001 (***). The lines within the box plots indicate the medians, and the lower and upper boundaries of the boxes indicate the 25th and 75th percentiles, respectively. Whiskers above and below the boxes correspond to 1.5 times the interquartile range (IQR) above and below the 25th and 75th percentiles, respectively

We found sex-specific differences in the community richness as measured by two different metrics: Males have a lower observed number of OTUs (LM Sex [M], *β* =  − 0.76 ± 0.32, 95% CI [− 1.40 to − 0.11], *p* = 0.022) (Fig. 3a) and Chao 1 (LM Sex [M], *β* =  − 0.85 ± 0.33, 95% CI [− 1.50 to − 0.19], *p* = 0.012) (Fig. 3b). However, we did not find any significant differences in the Shannon diversity index, which measures the diversity and evenness of the microbial communities, between males and females (LM Sex [M], *β* =  − 0.19 ± 0.24, 95% CI [− 0.66 to 0.29], *p* = 0.435) (Fig. 3c).

**Fig. 4:**
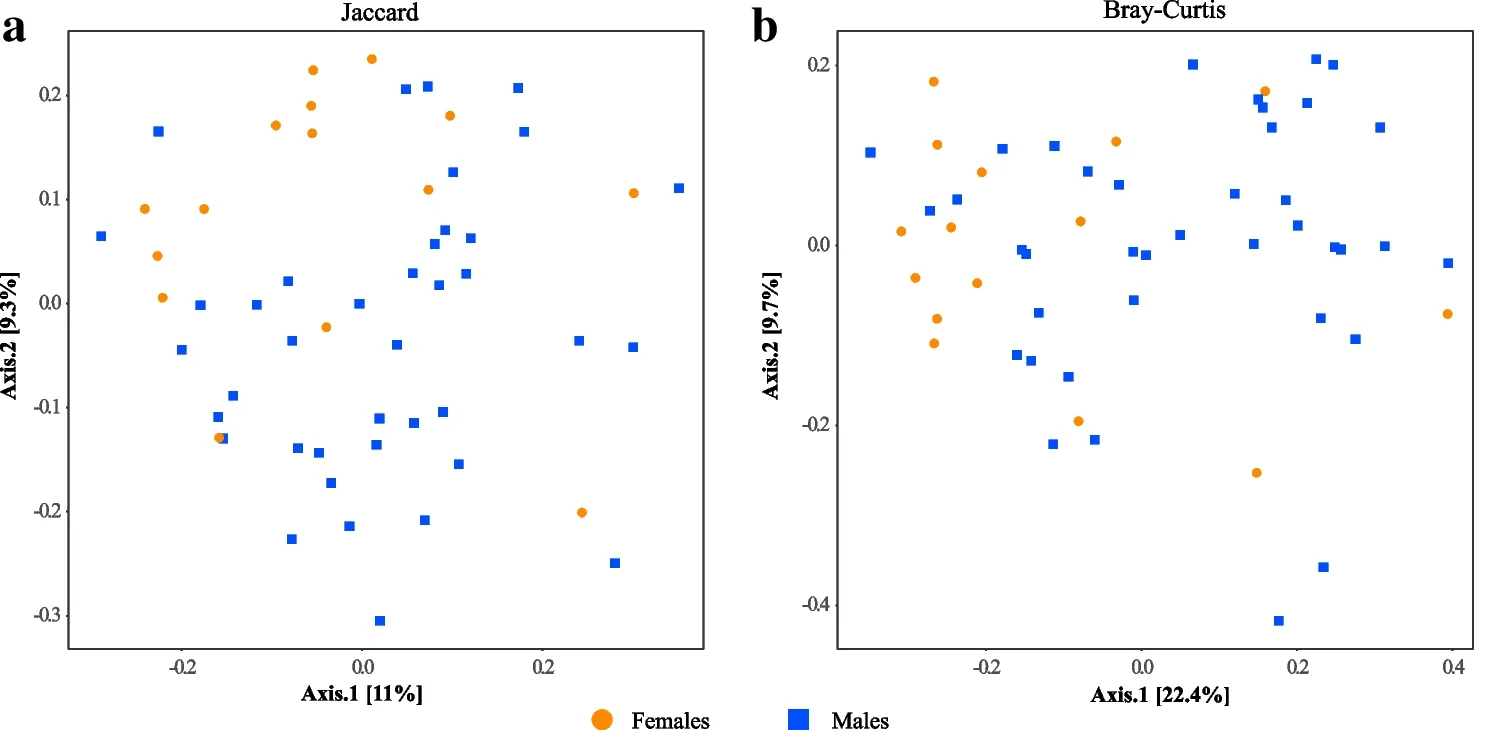
Principal coordinate analysis plots of the dissimilarities of wing sac microbiota of males and females. Distances were computed using the **a** Jaccard and **b** Bray–Curtis dissimilarity index

Microbial community composition also differed between males and females. Although the most abundant microbial family was *Moraxellaceae* in both males (44.70%) and females (38.46%), the most abundant families exhibited prominent differences between sexes (Fig. 2). In females, the most abundant microbial families were *Pseudomonadaceae* (7.69%), *Enterobacteriaceae* (7.24%), *Weeksellaceae* (7.17%), *Aeromonadaceae* (6.24%), and *Yersiniaceae* (6.05%) (Fig. 2a). In males, the communities were dominated by *Erwiniaceae* (12.36%), *Staphylococcaceae* (12.00%), *Enterobacteriaceae* (7.87%), *Xanthomonadaceae* (7.045%), and *Enterococcacea*e (5.54%) (Fig. 2b). When we statistically tested the observed compositional differences between the sexes using PERMANOVA, we found slight but statistically significant differences in the models based on Jaccard (*R*^2^ = 0.027; *p* = 0.042) and Bray–Curtis (*R*^2^ = 0.041; *p* = 0.01) resemblance matrices. A minimal compositional overlap between males and females was also visually supported by PCoA plots generated based on these two resemblance matrices (Fig. 4). Nevertheless, the sex-specific differences were not evident in the PERMANOVA models based on unweighted (*R*^2^ = 0.023; *p* = 0.233) and weighted UniFrac resemblance (*R*^2^ = 0.028; *p* = 0.162). PERMDISP analyses did not reveal any statistical difference in the homogeneity of group dispersion between males and females (all *p* values obtained from permutes were larger than 0.05), indicating that the significant PERMANOVA results were not caused by differences in dispersion among the groups.

**Fig. 5:**
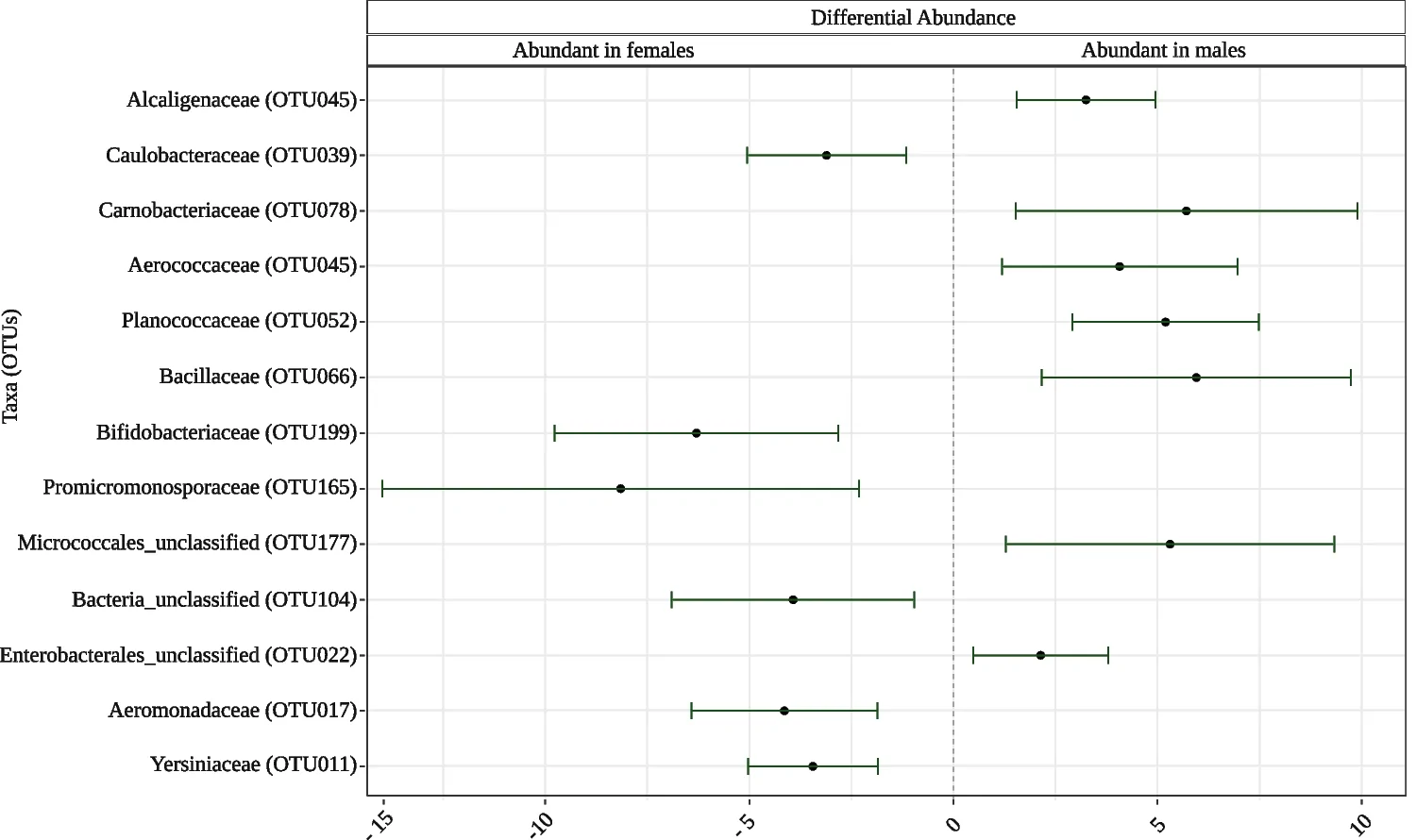
Differentially more abundant operational taxonomic units (OTUs) in males and females were determined using beta-binomial regression models in the Corncob package. The family-level taxonomy of each corresponding OTU is shown

We also identified the differentially abundant OTUs between samples collected from males and females using beta-binomial regression models and controlling for differential variability across sexes. Overall, we found 13 differentially abundant OTUs (Fig. 5, see Supplementary Table S1 for finer taxonomic assignments). Of these, six were significantly more abundant in female hosts. Notably, although males have overall lower microbial richness, seven OTUs were significantly more abundant in this group. Two OTUs showing a higher abundance in males belong to the microbial families containing lactic acid bacteria responsible for fermentation, *Aerococcaceae* and *Carnobacteriaceae* [47]. Furthermore, one of the OTUs with increased abundance in males belongs to the Enterobacterales order. Some of the species in this taxon are known to produce volatile organic compounds that contribute to the smell of cheese [48]. One of the OTUs belongs to the *Bacillus* genus of *Bacillaceae* family (Supplementary Table S1). Some *Bacillus* species are known to break down malodorous volatile organic compounds [49, 50]. One of the OTUs exhibiting an increased abundance in males belongs to the Micrococcales order, which contains some antimicrobial-producing bacteria [51].

## Microbial Communities Did Not Differ Between the Populations

The linear models did not reveal any significant differences in any of the alpha diversity measures between the two populations (Population [Palo Verde]; observed number of OTUs: *β* =  − 0.06 ± 0.29, 95% CI [− 0.63 to 0.52], *p* = 0.848); Chao 1: *β* = 0.02 ± 0.29, 95% CI [− 0.57 to 0.61], *p* = 0.934); Shannon’s diversity index: *β* = 0.34 ± 0.21, 95% CI [− 0.09 to 0.77], *p* = 0.114). PERMANOVA models did not show significant differences between the two colonies (all *p* values were larger than 0.05). Furthermore, we also tested whether samples collected from spatially closer locations have more similar microbial communities than geographically distant ones by testing the correlations between the distance matrix of the geographical coordinates of the sampling based on the Haversine distances and microbial resemblance matrices. Based on the Mantel test, there was no correlation between spatial proximity and microbial composition (all *p* values were larger than 0.05).

## Discussion

In our conceptual replication study, we repeated the study by Voigt and colleagues [15], which demonstrated sex-specific differences in the microbiota in the sexually selected scent organ of *S. bilineata* by adopting some methodological incremental improvements. Complementary to the original study, which relied on culture-dependent identification techniques, we employed a culture-independent molecular method, 16 s rRNA gene sequencing, and advanced statistical method to characterise wing-sac microbiota of *S. bilineata*. Unsurprisingly, our study identified more microbial taxa (277 versus 40) than the one of Voigt and colleagues [15], corroborating that culture-dependent methods can identify only a small proportion of the symbiotic bacteria that can be identified by 16 s rRNA gene sequencing. Nevertheless, our findings are consistent with the original study: wing-sac microbiota of *S. bilineata* exhibit sex-specific differences.

In line with the original study, microbial richness is lower in wing sacs of males than wing sac rudiments of females. In contrast to these findings, in wild spotted hyenas (*Crocuta crocuta*) [52] and white-tailed deer (*Odocoileus virginianus*) [53], males have richer scent gland microbiota than females. Considering that the wing sacs of the males are filled with potentially nutrient-rich excretions and are more humid than female rudiments, one can expect a higher microbial richness. Therefore, the observed sex-specific patterns in greater sac-winged bats cannot be attributable to morphological differences in the antebrachial wing membrane alone. Voigt and colleagues [15] proposed the male-specific behaviour of wing sac cleaning and refilling as one of the potential explanations for controlling microbial growth. During the first phase of this time-consuming and energetically costly ritual, males take up some urine into their mouths and then lick their wing sacs [24, 28–30]. Saliva is known to contain antimicrobial peptides [54] and can potentially inhibit microbial growth. Furthermore, urine is proposed to possess antimicrobial properties due to its hypertonic nature with a low pH and high concentrations of urea, which collectively deter the growth of most bacteria [55, 56].

Furthermore, we observed an increased abundance of operational taxonomic units (OTUs) belonging to the Micrococcales order in males. Some species in this taxon can produce antimicrobial substances inhibiting the growth of numerous bacteria [51], suggesting that regulation of this complex ecosystem might involve microbe-microbe interactions. Rojas-Gätjens and colleagues [51] suggested that Micrococcales species residing in the sloth fur could control hair microbiota in two sloth species.

Consistent with the original study, we also found slight sex-specific compositional differences in the scent organ microbiota. This finding is also in line with the previous studies showing sex-specific compositional differences in scent gland microbiota of wild spotted hyenas [52], meerkats (*Suricata suricatta*) [12], and owl monkeys (*Aotus nancymaae*) [57]. Among the OTUs exhibiting higher abundance in males, some belong to taxa containing potential odour producers, suggesting a potential role of microbially produced volatiles in the male scent profile [15]. One of the OTUs that are more abundant in males belongs to the *Bacillus* genus. Strikingly, some members of this taxon are known to break down malodorous volatile organic compounds [49, 50]. Nevertheless, it is important to note that the production of odours by bacteria is a complex process influenced by various factors, including the specific strain, environmental conditions, and the presence of substrates or nutrients.

In conclusion, the scarcity of replication studies, particularly in certain fields like ecology, hinders incremental progress and challenges scientific practice integrity. In this respect, our conceptual replication study investigating sex-specific differences in the wing-sac microbiota of *S. bilineata* fills an important gap. Employing modern molecular techniques and advanced statistical methods, we validated the original study’s findings. Our results add to the existing knowledge on the potential impact of microbially produced volatiles in shaping the scent profiles of male wing-sac bats, underlining the importance of replication efforts in corroborating scientific findings. Furthermore, our research opens new windows to study potential mechanisms behind the sex-specific regulation of this complex ecosystem and the role of microbial symbionts on the sexual selection of their hosts. We hope our study also encourages the scientific community to promote a culture that values replication studies as integral to the scientific process.

## Supplementary Information

Below is the link to the electronic supplementary material.

Supplementary file1 (PDF 19 KB)

## Data Availability

The datasets generated during the current study can be found in the European Nucleotide Archive repository, Project ID: PRJEB67961. The code used in the analyses is available in the GitHub repository at. https://github.com/AnnaAntonatouPap/-Bats-Bacteria-and-Bat-Smell-V.2.0-Repeatable-Sex-specific-Differences-in-Scent-Organ-Microbiota

## References

[1] Schmidt S (2009) Shall we Really do it Again? The powerful concept of replication is neglected in the social sciences. Rev Gen Psychol 13:90–100

[2] Zwaan RA, Etz A, Lucas RE, Donnellan MB (2018) Making replication mainstream. Behav Brain Sci 41:e120 10.1017/S0140525X17001972 29065933

[3] Nakagawa S, Parker TH (2015) Replicating research in ecology and evolution: feasibility, incentives, and the cost-benefit conundrum. BMC Biol 13:88 26510635 10.1186/s12915-015-0196-3 PMC4624660

[4] Albone ES, Perry GC (1976) Anal sac secretion of the red fox, Vulpes vulpes; volatile fatty acids and diamines: implications for a fermentation hypothesis of chemical recognition. J Chem Ecol 2:101–111

[5] Archie EA, Theis KR (2011) Animal behaviour meets microbial ecology. Anim Behav 82:425–436

[6] MartynL G, Nedwell DB, Smith RM (1974) An analysis of the contents of the anal scent pockets of Herpestes auropunctatus (Carnivora: Viverridae). J Zool 172:389–99

[7] Carthey AJR, Gillings MR, Blumstein DT (2018) The extended genotype: microbially mediated olfactory communication. Trends Ecol Evol 33:885–894 30224089 10.1016/j.tree.2018.08.010

[8] Ezenwa VO, Williams AE (2014) Microbes and animal olfactory communication: where do we go from here? BioEssays 36:847–854 24986361 10.1002/bies.201400016

[9] Whittaker DJ, Slowinski SP, Greenberg JM, Alian O, Winters AD, Ahmad MM et al (2019) Experimental evidence that symbiotic bacteria produce chemical cues in a songbird. J Exp Biol 222 Pt 20:jeb202978 10.1242/jeb.202978 31537652

[10] Theis KR, Venkataraman A, Dycus JA, Koonter KD, Schmitt-Matzen EN, Wagner AP et al (2013) Symbiotic bacteria appear to mediate hyena social odors. Proc Natl Acad Sci U S A 110:19832–19837 24218592 10.1073/pnas.1306477110 PMC3856825

[11] Leclaire S, Jacob S, Greene LK, Dubay GR, Drea CM (2017) Social odours covary with bacterial community in the anal secretions of wild meerkats. Sci Rep 7:3240 28607369 10.1038/s41598-017-03356-x PMC5468246

[12] Leclaire S, Nielsen JF, Drea CM (2014) Bacterial communities in meerkat anal scent secretions vary with host sex, age, and group membership. Behav Ecol 25:996–1004

[13] Theis KR, Venkataraman A, Wagner AP, Holekamp KE, Schmidt TM (2016) Age-related variation in the scent pouch bacterial communities of striped hyenas (Hyaena hyaena). In: Schulte BA, Goodwin TE, Ferkin MH (eds) Chemical signals in vertebrates 13. Springer International Publishing, Cham, pp 87–103

[14] Ma R, Zheng W, Guo J, Hou R, Huang H, Xue F et al (2022) Symbiotic microbiota and odor ensure mating in time for giant pandas. Front Microbiol 13:1015513 36466630 10.3389/fmicb.2022.1015513 PMC9712809

[15] Voigt CC, Caspers B, Speck S (2005) Bats, bacteria, and bat smell: sex-specific diversity of microbes in a sexually selected scent organ. J Mammal 86:745–749

[16] Bradbury JW, Emmons LH (1974) Social organization of some Trinidad bats. Z Tierpsychol 36:137–183 4478737 10.1111/j.1439-0310.1974.tb02130.x

[17] Voigt CC, von Helversen O, Michener R, Kunz TH (2001) The economics of harem maintenance in the sac-winged bat, Saccopteryx bilineata (Emballonuridae). Behav Ecol Sociobiol 50:31–36

[18] Voigt CC, Schwarzenberger F (2008) Reproductive endocrinology of a small tropical bat (female Saccopteryx bilineata; Emballonuridae) monitored by fecal hormone metabolites. J Mammal 89:50–57

[19] Tannenbaum (1975) Reproductive strategies in the white-lined bat (PhD dissertation). Ithaca, New York, Cornell University

[20] Heckel G, von Helversen O (2002) Male tactics and reproductive success in the harem polygynous bat Saccopteryx bilineata. Behav Ecol 13:750–756

[21] Voigt CC, Behr O, Caspers B, von Helversen O, Knörnschild M, Mayer F et al (2008) Songs, scents, and senses: sexual selection in the greater sac-winged bat, Saccopteryx bilineata. J Mammal 89:1401–1410

[22] Caspers B, Franke S, Voigt CC (2008) The wing-sac odour of male greater sac-winged bats Saccopteryx bilineata (Emballonuridae) as a composite trait: seasonal and individual differences. In: Hurst JL, Beynon RJ, Roberts SC, Wyatt TD (eds) Chemical signals in vertebrates 11. Springer, New York, NY, pp 151–160

[23] Schneeberger K, Schulze M, Scheffler I, Caspers BA (2021) Evidence of female preference for odor of distant over local males in a bat with female dispersal. Behav Ecol 32:657–661

[24] Caspers B, Wibbelt G, Voigt CC (2009) Histological examinations of facial glands in Saccopteryx bilineata (Chiroptera, Emballonuridae), and their potential use in territorial marking. Zoomorphology 128:37–43

[25] Caspers BA, Schroeder FC, Franke S, Voigt CC (2011) Scents of adolescence: the maturation of the olfactory phenotype in a free-ranging mammal. PLoS ONE 6:e21162 21738615 10.1371/journal.pone.0021162 PMC3124483

[26] Schneeberger K, Voigt CC, Müller C, Caspers BA (2016) Multidimensionality of chemical information in male greater sac-winged bats (Saccopteryx bilineata). Front Ecol Evol 4:86

[27] Scully WM, Fenton MB, Saleuddin AS (2000) A histological examination of the holding sacs and glandular scent organs of some bat species (Emballonuridae, Hipposideridae, Phyllostomidae, Vespertilionidae, and Molossidae). Can J Zool 78:613–623

[28] Voigt CC (2002) Individual variation in perfume blending in male greater sac-winged bats. Anim Behav 63:907–913

[29] Voigt CC, Heckel G, Mayer F (2005) Sexual selection favours small and symmetric males in the polygynous greater sac-winged bat Saccopteryx bilineata (Emballonuridae, Chiroptera). Behav Ecol Sociobiol 57:457–464

[30] Voigt CC, von Helversen O (1999) Storage and display of odour by male Saccopteryx bilineata (Chiroptera, Emballonuridae). Behav Ecol Sociobiol 47:29–40

[31] Maraci Ö, Antonatou-Papaioannou A, Jünemann S, Castillo-Gutiérrez O, Busche T, Kalinowski J et al (2021) The gut microbial composition is species-specific and individual-specific in two species of estrildid finches, the Bengalese finch and the zebra finch. Front Microbiol 12 10.3389/fmicb.2021.619141 PMC7933042 33679641

[32] Engel K, Sauer J, Jünemann S, Winkler A, Wibberg D, Kalinowski J et al (2018) Individual- and species-specific skin microbiomes in three different estrildid finch species revealed by 16S amplicon sequencing. Microb Ecol 76:518–529 29282519 10.1007/s00248-017-1130-8

[33] Magoč T, Salzberg SL (2011) FLASH: fast length adjustment of short reads to improve genome assemblies. Bioinformatics 27:2957–2963 21903629 10.1093/bioinformatics/btr507 PMC3198573

[34] Joshi NA, Fass JN. (2011) Sickle: a sliding-window, adaptive, quality-based trimming tool for FastQ files Version 1.33. https://github.com/najoshi/sickle

[35] Martin M (2011) Cutadapt removes adapter sequences from high-throughput sequencing reads. EMBnet.journal 17:10–2

[36] Schloss PD, Westcott SL, Ryabin T, Hall JR, Hartmann M, Hollister EB et al (2009) Introducing mothur: open-source, platform-independent, community-supported software for describing and comparing microbial communities. Appl Environ Microbiol 75:7537–7541 19801464 10.1128/AEM.01541-09 PMC2786419

[37] Edgar RC (2010) Search and clustering orders of magnitude faster than BLAST. Bioinformatics 26:2460–2461 20709691 10.1093/bioinformatics/btq461

[38] Quast C, Pruesse E, Yilmaz P, Gerken J, Schweer T, Yarza P et al (2013) The SILVA ribosomal RNA gene database project: improved data processing and web-based tools. Nucleic Acids Res 41:D590–D596 23193283 10.1093/nar/gks1219 PMC3531112

[39] R Core Team (2020) R: a language and environment for statistical computing. https://www.r-project.org/index.html

[40] DeSantis TZ, Hugenholtz P, Keller K, Brodie EL, Larsen N, Piceno YM et al (2006) NAST: a multiple sequence alignment server for comparative analysis of 16S rRNA genes. Nucleic Acids Res 34 Web Server issue:W394–9 10.1093/nar/gkl244 PMC1538769 16845035

[41] Shannon CE (1948) A mathematical theory of communication. Bell Syst Tech J 27:379–423

[42] Wickham H (2009) ggplot2: elegant graphics for data analysis. New York, NY: Springer New York

[43] Oksanen J, Blanchet FG, Friendly M, Kindt R, Legendre P, McGlinn D et al (2019) Package ‘vegan.’ Community ecology package, version 2

[44] Anderson MJ (2001) A new method for non-parametric multivariate analysis of variance. Austral Ecol 26:32–46

[45] Martin BD, Witten D, Willis AD (2020) Modeling microbial abundances and dysbiosis with beta-binomial regression. Ann Appl Stat 14:94–115 32983313 10.1214/19-aoas1283 PMC7514055

[46] Benjamini Y, Hochberg Y (1995) Controlling the false discovery rate: a practical and powerful approach to multiple testing. J Roy Stat Soc: Ser B (Methodol) 57:289–300

[47] Zheng J, Wittouck S, Salvetti E, Franz CMAP, Harris HMB, Mattarelli P et al (2020) A taxonomic note on the genus Lactobacillus: description of 23 novel genera, emended description of the genus Lactobacillus Beijerinck 1901, and union of Lactobacillaceae and Leuconostocaceae. Int J Syst Evol Microbiol 70:2782–2858 32293557 10.1099/ijsem.0.004107

[48] Ritschard JS, Van Loon H, Amato L, Meile L, Schuppler M (2022) High prevalence of Enterobacterales in the smear of surface-ripened cheese with contribution to organoleptic properties. Foods 11:361 35159512 10.3390/foods11030361 PMC8834058

[49] Nowocień K, Sokołowska B (2022) Bacillus spp. as a new direction in biocontrol and deodorization of organic fertilizers. AIMSES 9:95–105

[50] Ushida K, Hashizume K, Miyazaki K, Kojima Y, Takakuwa S (2003) Isolation of Bacillus sp. as a volatile sulfur-degrading bacterium and its application to reduce the fecal odor of pig. Asian-Australasian J An Sci 16:1795–8

[51] Rojas-Gätjens D, Valverde-Madrigal KS, Rojas-Jimenez K, Pereira R, Avey-Arroyo J, Chavarría M (2022) Antibiotic-producing Micrococcales govern the microbiome that inhabits the fur of two- and three-toed sloths. Environ Microbiol 24:3148–3163 35621042 10.1111/1462-2920.16082

[52] Rojas CA, Holekamp KE, Winters AD, Theis KR (2020) Body site-specific microbiota reflect sex and age-class among wild spotted hyenas. FEMS Microbiol Ecol 96:fiaa007 10.1093/femsec/fiaa007 31926016

[53] Gassett JW, Dasher KA, Miller KV, Osborn DA, Russell SM (2000) White-tailed deer tarsal glands : sex and age-related variation in microbial flora. 64:371–8

[54] Vila T, Rizk AM, Sultan AS, Jabra-Rizk MA (2019) The power of saliva: antimicrobial and beyond. PLoS Pathog 15:e1008058 31725797 10.1371/journal.ppat.1008058 PMC6855406

[55] Chambers ST, Lever M (1996) Betaines and urinary tract infections. Nephron 74:1–10 8883013 10.1159/000189274

[56] Kucheria R, Dasgupta P, Sacks SH, Khan MS, Sheerin NS (2005) Urinary tract infections: new insights into a common problem. Postgrad Med J 81:83–86 15701738 10.1136/pgmj.2004.023036 PMC1743204

[57] Bowen M, Miles C, Hegseth R, Anderson CM, Brandon CS, Langford ML et al (2021) The potential interplay between the glandular microbiome and scent marking behavior in owl monkeys (Aotus nancymaae). Am J Primatol 83:e23324 34492124 10.1002/ajp.23324

