# Supplementary material for: Bats, Bacteria, and Bat Smell V.2.0: Repeatable Sex-Specific Differences in Scent Organ Microbiota
Source: Microb Ecol. 2024 Mar 26;87:55. doi: 10.1007/s00248-024-02368-1 (PMC10965658; doi:10.1007/s00248-024-02368-1)
Supplement: Supplementary file 1 [file 248_2024_2368_MOESM1_ESM.pdf]

**Supplementary Information 2. Differentially abundant OTUs between males and females**

| OTU No | padj. | Taxonomy | More abundant |
|---|---|---|---|
| OTU011 | <0.001 | Bacteria_Proteobacteria_Gammaproteobacteria_Enterobacterales_Yersiniaceae_Serratia | Females |
| OTU017 | 0.003 | Bacteria_Proteobacteria_Gammaproteobacteria_Aeromonadales_Aeromonadaceae_Aeromonas | Females |
| OTU039 | 0.014 | Bacteria_Proteobacteria_Alphaproteobacteria_Caulobacterales_Caulobacteraceae_Brevundimonas | Females |
| OTU104 | 0.037 | Bacteria_unclassified | Females |
| OTU119 | 0.004 | Bacteria_Actinobacteriota_Actinobacteria_Bifidobacteriales_Bifidobacteriaceae_Bifidobacteriaceae_unclassified | Females |
| OTU165 | 0.032 | Bacteria_Actinobacteriota_Actinobacteria_Micrococcales_Promicromonosporaceae_Isoptericola | Females |
| OTU022 | 0.039 | Bacteria_Proteobacteria_Gammaproteobacteria_Enterobacterales_Enterobacterales_unclassified | Males |
| OTU045 | 0.032 | Bacteria_Firmicutes_Bacilli_Lactobacillales_Aerococcaceae_Aerococcus | Males |
| OTU047 | 0.002 | Bacteria_Proteobacteria_Gammaproteobacteria_Burkholderiales_Alcaligenaceae_Alcaligenaceae_unclassified | Males |
| OTU052 | <0.001 | Bacteria_Firmicutes_Bacilli_Bacillales_Planococcaceae_Lysinibacillus | Males |
| OTU066 | 0.014 | Bacteria_Firmicutes_Bacilli_Bacillales_Bacillaceae_Bacillus" | Males |
| OTU078 | 0.034 | Bacteria_Firmicutes_Bacilli_Lactobacillales_Carnobacteriaceae_Desemzia | Males |
| OTU177 | 0.037 | Bacteria_Actinobacteriota_Actinobacteria_Micrococcales_Micrococcales_unclassified_Micrococcales_unclassified | Males |

**Article title:** Bats, Bacteria and Bat Smell V.2.0: Repeatable Sex-specific Differences in Scent Organ Microbiota

**Journal Name:** Microbial Ecology

**Author information**

Öncü Maraci[1,2*], Anna Antonatou-Papaioannou[3-4], Sebastian Jünemann[5-6], Karin Schneeberger[7], Michael Schulze[7], Ingo Scheffler[8], Barbara A. Caspers[1,2]

Schneeberger[7], Michael Schulze[7], Ingo Scheffler[8], Barbara A. Caspers[1,2]

[1]Department of Behavioural Ecology, Bielefeld University, Konsequenz 45, 33619 Bielefeld, Germany.

[2]Joint Institute for Individualisation in a Changing Environment (JICE), University of Münster and Bielefeld University, Germany.

[3]Evolutionary Biology, Bielefeld University, Universitätsstrasse 25, 33615, Bielefeld, Germany.

[4]Institute of Biology-Zoology, Freie Universität Berlin, Köning-Luise-Str. 1-3, 14195, Berlin.

[5]Faculty of Technology, Bielefeld University, Universitätsstrasse 25, 33615, Bielefeld, Germany.

[6]Institute for Bio- and Geosciences, IBG-5, Research Center Jülich, Bielefeld University, Universitätsstrasse 27, 33615, Bielefeld, Germany.

[7]Animal Ecology, Institute of Biochemistry and Biology, University of Potsdam, Maulbeerallee 1, 14469 Potsdam, Germany.

[8]Evolutionary Adaptive Genomics, Institute for Biochemistry and Biology, University of Potsdam, Karl-Liebknecht-Straße 24-26, 14476 Potsdam, Germany.

***Correspondence:** Öncü Maraci

E-mail: oncu.maraci@uni-bielefeld.de
