# Supplementary material for: Bats, Bacteria, and Bat Smell V.2.0: Repeatable Sex-Specific Differences in Scent Organ Microbiota
Source: Microb Ecol. 2024 Mar 26;87(1):55. doi: 10.1007/s00248-024-02368-1 (PMC10965658; doi:10.1007/s00248-024-02368-1)
Supplement: Supplementary file 1 — Supplementary file1 (PDF 19 KB) [file 248_2024_2368_MOESM1_ESM.pdf]

Supplementary Information 2. Differentially abundant OTUs between males and females

OTU No	padj.	Taxonomy	More abundant
OTU011	<0.001	Bacteria_Proteobacteria_Gammaproteobacteria_Enterobacterales_Yersiniaceae_Serratia	Females
OTU017	0.003	Bacteria_Proteobacteria_Gammaproteobacteria_Aeromonadales_Aeromonadaceae_Aeromonas	Females
OTU039	0.014	Bacteria_Proteobacteria_Alphaproteobacteria_Caulobacterales_Caulobacteraceae_Brevundimonas	Females
OTU104	0.037	Bacteria_unclassified	Females
OTU119	0.004	Bacteria_Actinobacteriota_Actinobacteria_Bifidobacteriales_Bifidobacteriaceae_Bifidobacteriaceae_unclassified	Females
OTU165	0.032	Bacteria_Actinobacteriota_Actinobacteria_Micrococcales_Promicromonosporaceae_Isoptericola	Females
OTU022	0.039	Bacteria_Proteobacteria_Gammaproteobacteria_Enterobacterales_Enterobacterales_unclassified	Males
OTU045	0.032	Bacteria_Firmicutes_Bacilli_Lactobacillales_Aerococcaceae_Aerococcus	Males
OTU047	0.002	Bacteria_Proteobacteria_Gammaproteobacteria_Burkholderiales_Alcaligenaceae_Alcaligenaceae_unclassified	Males
OTU052	<0.001	Bacteria_Firmicutes_Bacilli_Bacillales_Planococcaceae_Lysinibacillus	Males
OTU066	0.014	Bacteria_Firmicutes_Bacilli_Bacillales_Bacillaceae_Bacillus"	Males
OTU078	0.034	Bacteria_Firmicutes_Bacilli_Lactobacillales_Carnobacteriaceae_Desemzia	Males
OTU177	0.037	Bacteria_Actinobacteriota_Actinobacteria_Micrococcales_Micrococcales_unclassified_Micrococcales_unclassified	Males

Article title: Bats, Bacteria and Bat Smell V.2.0: Repeatable Sex-specific Differences in Scent Organ Microbiota

Journal Name: Microbial Ecology

Author information

Öncü Maraci[1,2*], Anna Antonatou-Papaioannou[3-4], Sebastian Jünemann[5-6], Karin Schneeberger[7], Michael Schulze[7], Ingo Scheffler[8], Barbara A. Caspers[1,2]

Schneeberger[7], Michael Schulze[7], Ingo Scheffler[8], Barbara A. Caspers[1,2]

[1]Department of Behavioural Ecology, Bielefeld University, Konsequenz 45, 33619 Bielefeld, Germany.

[2]Joint Institute for Individualisation in a Changing Environment (JICE), University of Münster and Bielefeld University, Germany.

[3]Evolutionary Biology, Bielefeld University, Universitätsstrasse 25, 33615, Bielefeld, Germany.

[4]Institute of Biology-Zoology, Freie Universität Berlin, Köning-Luise-Str. 1-3, 14195, Berlin.

[5]Faculty of Technology, Bielefeld University, Universitätsstrasse 25, 33615, Bielefeld, Germany.

[6]Institute for Bio- and Geosciences, IBG-5, Research Center Jülich, Bielefeld University, Universitätsstrasse 27, 33615, Bielefeld, Germany.

[7]Animal Ecology, Institute of Biochemistry and Biology, University of Potsdam, Maulbeerallee 1, 14469 Potsdam, Germany.

[8]Evolutionary Adaptive Genomics, Institute for Biochemistry and Biology, University of Potsdam, Karl-Liebknecht-Straße 24-26, 14476 Potsdam, Germany.

***Correspondence:** Öncü Maraci

E-mail: oncu.maraci@uni-bielefeld.de
